# Small vs. Large Suture Bite-to-Stitch Interval for Closure of Midline Celiotomy in Cats: A Biomechanical Study

**DOI:** 10.3389/fvets.2020.00206

**Published:** 2020-04-22

**Authors:** Fernando S. Reina Rodriguez, Joshua Milgram, Barbara M. Kirby

**Affiliations:** ^1^AÚNA Especialidades Veterinarias, Small Animal Surgery Service, Valencia, Spain; ^2^Koret School of Veterinary Medicine, The Hebrew University of Jerusalem, Rehovot, Israel; ^3^School of Veterinary Medicine, University College Dublin, Dublin, Ireland

**Keywords:** biomechanical testing, bursting strength, feline, hernia, laparotomy, suture pattern

## Abstract

**Objective:** The objective of this study was to compare the bursting strength and characterize the mode of failure of cranial and caudal midline celiotomies closed with 2 suture patterns and an absorbable monofilament suture material.

**Design:** Randomized, cadaveric, *ex- vivo* mechanical testing.

**Sample:** Feline cadavers (*n* = 32).

**Methods:** Specimens were randomized into two groups based on the closure technique (small 2 × 2 mm or large 5 × 5 mm suture-bite-stitch-interval [SBSI] groups). Cranial or caudal midline celiotomies, 7.5 cm long, were created. A custom-made polyurethane bladder was inserted into the abdomen, and the celiotomies were closed in a simple continuous pattern using 3-0 polydioxanone. The repair was loaded to failure by inflating the bladder with compressed air. Bursting strength and mode of failure were recorded. Effects of body weight, gender, thickness and width of linea alba, suture-bite-stitch-interval, and location of celiotomy were analyzed using a mixed model analysis and an independent t- test, with P &lt; 0.05 considered statistically significant.

**Results:** There was no difference in bursting strength between cranial and caudal celiotomies. Bursting strength was lower for celiotomies closed with a large SBSI (*P* = 0.003). Bursting strength was greater in males compared to females (*P* = 0.003). Twenty five specimens failed distant from celiotomy closure, while 4 failed by fascial tearing at the site of needle penetration. Failure by loosening of the suture line with intact knots only occurred in 3 caudal celiotomies closed with a large SBSI. Gender, body weight and SBSI accounted for 61.5% of variability in bursting strength (*P* = 0.005).

**Conclusions:** Small SBSI technique was mechanically superior to large SBSI when tested under these loading conditions.

**Clinical relevance:** Supraphysiological pressures were required to cause failure in all specimens. Both small and large SBSI may be clinically applicable for midline celiotomy closure in domestic cats.

## Introduction

Ventral midline celiotomy is one of the most common surgical approaches for abdominal surgery in small animals. Failure of the suture line used to close the celiotomy is a reported complication and is recognized clinically as incisional herniation, which may be acute or chronic, and may present either with or without eventration ((1), p. 355–361). Acute incisional hernias occur within days after surgery and are likely associated with inadequate surgical technique during closure ((1, 2), 98–102; (3), p. 57–63), inappropriate choice of suture material (1, 2) and trauma to the abdominal wall during the postoperative period (4). Chronic incisional hernias appear weeks to years after surgery (3) and are commonly identified in association with metabolic disease, immunosuppression, or development of surgical site infection (1, 2).

The complication rate for ventral midline celiotomy, including incisional hernia, appears to be very low in small animals (5); however, the consequences may be severe (4), particularly when accompanied by eventration (1, 4). Despite the reported low incidence, some authors have suggested that the true incidence of this postsurgical complication may be higher, as it is likely underreported (1, 6). This suggestion is supported by the results of a retrospective study of major abdominal evisceration in dogs and cats, where half of the dogs and all cats included in the study developed incisional hernias within 4 days after ovariohysterectomy (4). The data available in the literature about the true incidence of incisional hernia in small animals is not up to date, and likely does not accurately reflect the current situation due to improvements in surgical techniques and suture materials available.

An adequate method of wound closure, and optimal suture technique are important factors under the surgeon's control which may prevent incisional herniation (1). In people, the distance from the wound edges to where sutures enter and exit the tissue, and the distance between stitches is defined as the suture-bite-stitch-interval (SBSI). It has been shown that SBSI has an effect on the incidence of postsurgical incisional herniation in human patients (7–11). However, in small animals the SBSI is rarely reported or is based on personal experience rather than biomechanical tests ((5, 12), p. 405–413). According to previous recommendations in small animals, the stitch interval increases with body size and ranges from 3 to 12 mm; however, no information was provided about the suture bites (12). The only biomechanical study performed in small animals comparing two SBSI did not find differences between a small and a large SBSI technique; however differences were observed in the mode of failure of the specimens tested (13). Despite these findings, this study was limited by the use of discrete segments of ventral abdominal wall, which were unlikely to represent the entire abdominal wall musculature, and the use of uniaxial loading, which may be a poor simulation of the forces acting on a celiotomy closure during the postoperative period (13).

In the caudal third of the abdominal wall of dogs and cats, the internal leaf of the rectus sheath disappears and only the peritoneum covers the dorsal aspect of the rectus abdominus muscle at this level ((14), p. 153–156; (15), p. 224–227]. Biomechanical tests of the feline ventral abdominal wall have shown that the region caudal to the umbilicus has lower load to failure when compared to the umbilical region and the region cranial to the umbilicus, possibly due to differences in the thickness of the linea alba between the 3 regions (13). Based on anatomical (14) and biomechanical findings (13), there appears to be an inherent weakness in the body wall in the region caudal to the umbilicus in domestic cats.

The aim of this study was to compare bursting strength between cranial and caudal midline celiotomies closed with two SBSI techniques. We hypothesized that caudal celiotomies, which include the region caudal to the umbilicus, would have a decreased bursting strength relative to cranial celiotomies. We also hypothesized that celiotomies closed with a small SBSI technique, would have increased bursting strength compared to large SBSI.

## Materials and Methods

### Specimen Population

This study was performed using adult feline cadavers, euthanized for reasons unrelated to this study. Specimens were provided by donation centers and information available included estimated age, ongoing medical treatment prior to euthanasia, and date of euthanasia. Ethical approval from the institutional Animal Research Ethics Committee was obtained prior to performing this study. Cadavers were included if there was no visual evidence of pathology of the abdominal wall from either local or systemic disease (e.g., obesity, cachexia, abdominal surgery, abdominal trauma, or neoplasia).

### Specimen Preparation

Cadavers were placed into a freezer within 30 min of euthanasia and stored at −80° C until testing. Twenty four hours prior to testing, cadavers were removed from the freezer and allowed to thaw at room temperature. Once thawed, the body weight (BW), sex, and breed were recorded. All cats were positioned in dorsal recumbency, and after clipping the hair, the ventral body wall was visually inspected to identify any criteria warranting exclusion from the study.

**Figure 1:**
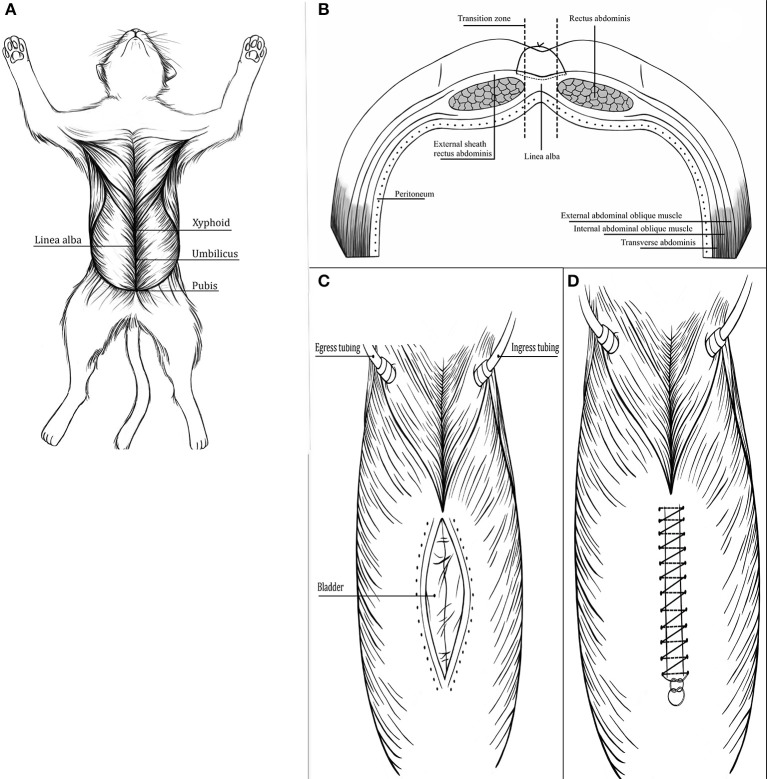
**(A)** Cat in dorsal recumbency after reflecting the skin and subcutaneous tissue from the xiphoid to the pubis to expose the entire ventral abdominal wall and linea alba. **(B)** Diagram (transverse view) showing suture placement and layers included in celiotomy closure. **(C)** Custom made bladder placed in the abdominal cavity after celiotomy and connected to egress and ingress tubes. **(D)** Celiotomy closure completed.

The entire length of the linea alba and ventral body wall cranial and caudal to it were exposed by incising the skin of the ventral abdomen, and reflecting the subcutaneous soft tissues (Figure 1A). Once exposed, the width of the linea alba was measured with digital calipers (Fisherbrand™ Traceable™ Digital Calipers, Thermo Fisher Scientific) at the cranial abdominal (CrA), umbilical (U), and caudal abdominal (CdA) regions. At each location, the width of the linea alba was measured three times and the average of these measurements was recorded.

### Study Groups

Specimens were randomly assigned to 2 groups based on location of the celiotomy using a statistical software package (SPSS Statistics, Version 20). In group 1 (cranial celiotomy) a 7.5 cm long full thickness incision was made in the linea alba starting at the umbilicus and extending cranially toward the xiphoid cartilage (CrA). In group 2 (caudal celiotomy) a 7.5 cm long full thickness incision was made in the linea alba starting at the umbilicus and extending caudally toward the pubis (CdA).

**Figure 2:**
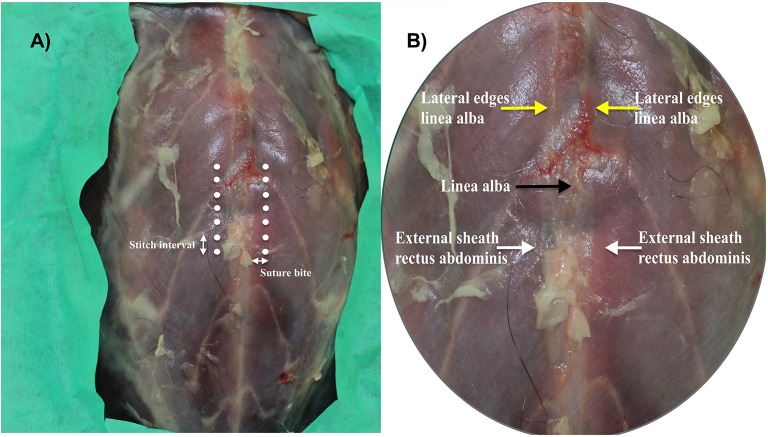
**(A)** Specimen showing suture-bite and stitch intervals (SBSI). **(B)** Close view of the same specimen showing the linea alba (black arrow), the lateral edges of the linea alba (yellow arrows) and the external leaf of the rectus abdominis (white arrows). At the microscopic level, the area between the lateral edges of the linea alba and the external sheath of the rectus abdominis is the transition zone (16).

Two suture patterns for celiotomy closure were defined based on distance of the location of needle penetration from the lateral edge of the linea alba [suture bite (SB)] and distance between stitches [stitch interval (SI)]. The suture patterns (SBSI) were designated as small 2 × 2 or large 5 × 5, indicating 2 or 5 mm distance of suture bites from the lateral edge of the linea alba, and 2 or 5 mm distance between stitches (Figure 2A). Suture bites were measured from the lateral edge of the linea alba to engage the transition zone between the linea alba and the rectus sheath, as previously described (13, 16) (Figures 1B, 2B). Distances for each SBSI were marked on hectograph paper using a marker and a ruler. A template was drawn with the linea alba centered, and points marking distance (mm) between stitches, and distance (mm) from the lateral edge of the linea alba. The template was placed in contact with the abdominal wall and the location of each needle penetration was maked on the abdominal wall prior to incising the linea alba to ensure accurate spacing (Figure 2A). Specimens from group 1 and group 2 were randomly selected with the same statistical software package used previously, for celiotomy closure using the small or the large SBSI technique.

After incising the linea alba, the thickness of the linea alba was measured on each specimen with digital calipers, taking care not to compress the tissue during measurement. For cranial celiotomies, measurements were taken at the most cranial and caudal part of the incision, which corresponded to the CrA and U regions. For caudal celiotomies, measurements were taken at the most cranial and caudal extent of the incision, which corresponded to the U and CdA regions. At each location, the thickness of the linea alba was measured three times and the average measurement recorded.

### Biomechanical Testing

After celiotomy, a custom 12 × 20 cm rectangular, 12 L capacity, polyurethane bladder (New World Mfg Inc.) with integrated ingress and egress tubing, was inserted into the abdomen (Figure 1C). Care was taken to position the bladder flat along the ventral aspect of the abdominal wall. Stab incisions were made in the left and right 12th intercostal spaces to provide access for ingress and egress tubing. Ingress tubing was exteriorized through the left incision (Figure 1C) and attached to a 9 L capacity air compressor (SIL- EOL 9/30 silent compressor, MGF Compressors Srl), using a stainless steel adapter and extension set for recording invasive blood pressure (Small Bore extension set, MILA International Inc.). The compressor had manual air flow regulator and pressure switch (Condor MDR 2 air pressure switch, Condor Werker GmbH). Air flow was adjusted to deliver 10L/ min. Egress tubing was exteriorized through the right incision (Figure 2B) and connected to a digital manometer (PCE- 30 differential pressure manometer, PCE Instruments Ltd.), using a silicon tube (SS- AZ silicon tubes, PCE Instruments Ltd.), an extension set (Small Bore extension set, MILA International Inc.), and a barbed adapter (Male Luer Lock Christmas tree connector, MILA International Inc.). The digital manometer had a data logging system to record pressures in real time. Data was transferred to a laptop and a pressure- time curve was generated using dedicated software (SOFTP- AZ, PCE Instruments Ltd.).

Once the bladder was placed and the tubes connected, celiotomies were closed with USP 3-0 polydioxanone (PDS II®, Ethicon Inc.) on a 26 mm SH taper needle. Care was taken to include only the external sheath of the rectus abdominis muscle fascia in each bite (Figure 1B). A simple continuous pattern was used with square knots of 5 throws at the beginning (caudal knot) and 6 throws at the end (cranial knot) (Figure 1D), according to recommendations for this suture material and suture pattern (17). Suture ends were cut 3 mm from knots (17), and the length of the ears were confirmed with digital calipers. Closure was performed in a caudal to cranial direction and only to appose the edges of the celiotomy without compressing the tissue with each bite.

For each specimen, the number of stitches used to close the celiotomy and the surgical time, defined as the time required to complete the celiotomy closure, were recorded. In addition, the length of suture material used for closure was calculated by subtracting the remaining length of suture material (measured with digital calipers) from the initial suture material length as recorded on the suture packet. The resultant value was used to calculate the suture length to wound length ratio (SL:WL). This ratio reflects the length of suture material used to close the incision, the length of the incision, and, together with the suture bite and stitch interval, the number of stitches used for closure (18).

All specimens were tested within 24 h of being thawed, remaining immersed in a phosphate buffered saline bath at 4°C prior to testing.

The bladder was inflated with compressed room air until failure, which was defined as a sudden decrease in the slope of the pressure- time curve, and corresponded to rupture of the abdominal wall or suture line during testing. The maximum pressure (expressed in mm of Hg) required to burst the suture wound or cause tissue failure was defined as bursting strength (19).

The mode of failure for each specimen was recorded using a digital camera (Olympus VG- 160, Olympus Corporation). All tests were performed and interpreted by a single investigator.

### Statistical Analysis

Data was analyzed with a statistical software package (SPSS Statistics, Version 20). A test for normality was performed for all variables. Spearman's rank coefficient was calculated to evaluate the correlation between body weight (BW), sex, thickness and width of the linea alba, and bursting strength.

Independent *t*-test with Welch's correction was performed to compare bursting strength between groups and between sex. Mixed model linear analysis was used to evaluate the effect of SBSI and location of celiotomy (repeat measures/fixed effects) on bursting strength (dependent variable). Sex, BW, thickness and width of linea alba were the subjects/random effects in the statistical model. Stepwise regression was performed to remove non-significant variables from the model with *P* < 0.05 considered statistically significant. Regression analysis was performed to evaluate the influence of independent variables on bursting strength. One- way ANOVA for the final statistical model was performed to compare bursting strength between groups followed by a *post-hoc* analysis, with *P* < 0.05 considered significant.

## Results

### Specimens

Thirty-five adult cat cadavers were evaluated for inclusion in the study. Two cats were excluded, 1 with evidence of blunt trauma affecting the abdominal and thoracic walls, and 1 with hemoabdomen and hematomas in the abdominal musculature. One specimen was used as a pilot and was not included in the statistical analysis. Thirty- two cats (*n* = 32) were included in the study. There were 17 females (5 entire, 12 spayed), and 15 males (9 entire, 6 castrated). Breeds included 23 domestic shorthairs, 7 domestic longhairs, 1 Persian, and 1 Russian Blue.

### Study Groups

Data for the study groups is summarized in Table 1. Sixteen specimens had cranial celiotomy of which 8 were closed with a small 2 × 2 SBSI, and 8 with a large 5 × 5 SBSI. Sixteen specimens had caudal celiotomy of which 8 were closed with a small SBSI, and 8 with a large SBSI. Nine males had cranial celiotomy and 6 caudal celiotomy. Seven females had cranial celiotomy and 10 caudal celiotomy. Eight males had small 2 × 2 SBSI celiotomy closure and 7 large 5 × 5 SBSI closure. Seven females had small SBSI celiotomy closure and 10 large SBSI closure.

**Table 1:** Summary of the data for the 32 specimens.

**Group**	**Sex**	BW^*^ (kg)	**Thickness linea alba (mm)**			**Width linea alba (mm)**			**Number stitches**	**Surgical time (min)**	**SL:WL**	**Bursting strength (mmHg)**
			**CrA^†^**	**U^‡^**	**CdA^§^**	**CrA^†^**	**U^‡^**	**CdA^§^**				
Small 2 × 2 SBSI^**^ cranial celiotomy	Males (n = 4)	3.9 (2.8–5.9)	1.17 ± 0.22	0.95 ± 0.08		1.7 ± 0.48	3.1 ± 0.55	1 ± 0.33	43 ± 5.4	9 ± 0.96	5 ± 0.33	639 ± 120
	Females (*n* = 4)	2.5 (2.1–3.2)	1.01 ± 0.43	0.55 ± 0.22		1.8 ± 0.86	2.8 ± 1.2	1.1 ± 0.52	43 ± 5.2	10 ± 0.86	5.3 ± 0.24	467 ± 67
	Total (*n* = 8)	3 (2.1–5.9)	1.1 ± 0.33	0.75 ± 0.26		1.7 ± 0.65	2.9 ± 0.87	1 ± 0.4	43 ± 4.9	9.6 ± 1.1	5.2 ± 0.32	553 ± 129
Small 2 × 2 SBSI^**^ caudal celiotomy	Males (n= 4)	4.7 (2.9–4.8)		1.3 ± 0.86	0.85 ± 0.38	1.7 ± 0.66	4.1 ± 0.67	1.2 ± 0.72	39 ± 3.1	7.8 ± 1	4.9 ± 0.53	632 ± 81
	Females (*n* = 4)	2.9 (2.4–3.5)		0.86 ± 0.25	0.62 ± 0.32	2.1 ± 0.67	4.4 ± 3.3	1.2 ± 0.33	39 ± 1.9	7.3 ± 1.6	5.1 ± 0.79	517 ± 72
	Total (*n* = 8)	3.3 (2.4–4.8)		1.1 ± 0.63	0.74 ± 0.34	1.9 ± 0.65	4.3 ± 2.2	1.2 ± 0.52	39 ± 2.4	7.5 ± 1.3	5 ± 0.63	575 ± 94
Large 5 × 5 SBSI^**^ cranial celiotomy	Males (*n* = 5)	3.1 (2.4–5.7)	1.4 ± 0.52	1.1 ± 0.3		1.8 ± 0.51	2.8 ± 0.65	0.95 ± 0.37	18 ± 1.6	4.7 ± 1.1	4.4 ± 0.31	539 ± 123
	Females (*n* = 3)	2.8 (2.6–3.8)	0.71 ± 0.2	0.6 ± 0.22		1.6 ± 0.4	2.8 ± 0.28	0.99 ± 0.26	18 ± 2.1	5.5 ± 0.25	4.3 ± 0.11	420 ± 26
	Total (*n* = 8)	3 (2.4–5.7)	1.2 ± 0.55	0.93 ± 0.38		1.7 ± 0.46	2.8 ± 0.52	0.97 ± 0.31	18 ± 1.7	5 ± 0.92	4.3 ± 0.25	495 ± 113
Large 5 × 5 SBSI^**^ caudal celiotomy	Males (*n* = 2)	4 (2.3–5.7)		0.88 ± 0.67	0.67 ± 0.4	3.9 ± 3.5	5.9 ± 4.7	1.1 ± 0.74	19 ± 0.71	5 ± 1.1	4.3 ± 0.15	520 ± 103
	Females (*n* = 6)	3.7 (2.6–4.7)		0.86 ± 0.47	0.69 ± 0.43	1.8 ± 0.38	3.3 ± 0.71	1.1 ± 0.18	19 ± 1.5	5.1 ± 1.1	4.3 ± 0.23	427 ± 81
	Total (*n* = 8)	3.7 (2.3–5.7)		0.87 ± 0.47	0.68 ± 0.39	2.3 ± 1.7	3.9 ± 2.2	1.1 ± 0.32	19 ± 1.3	5.1 ± 1	4.3 ± 0.2	451 ± 90

*Results expressed as mean ± SD except for body weight, expressed as median and range*.

* BW, Body weight;

† CrA, Cranial abdomen;

‡ U, Umbilicus;

§ CdA, Caudal abdomen;

¶ SL:WL, Suture length to wound length ratio;

** *SBSI, suture bite to stich interval*.

### Descriptive Statistics

Test for normality revealed all variables, except BW, were normally distributed. The median BW was 3.19 kg (range 2.12–5.86). The width of the linea alba (mean ± SD) was 1.9 ± 0.96 mm at the CrA (range 0.96–6.34), 3.48 ± 1.70 mm at the U (range 1.51–9.37), and 1.07 ± 0.39 mm at the CdA (range 0.49–2.24). The linea alba was widest at the U in all specimens (*P* < 0.0001). The thickness of the linea alba (mean ± SD) was 1.1 ± 0.44 mm at the CrA (range 0.49–2.1), 0.9 ± 0.45 mm at the U (range 0.3–2.5), and 0.71 ± 0.36 mm at the CdA (range 0.23–1.4). The thickness of the linea alba decreased gradually from cranial to caudal in all specimens (*P* = 0.02).

Mean ± SD number of stitches was 41 ± 4.13 (range 35–49) for 2 × 2 SBSI celiotomies, and 18.25 ± 1.53 (range 16–21) for 5 × 5 SBSI celiotomies. More stitches were used to close small SBSI celiotomies (*P* < 0.0001). Suture length to wound length ratio was ≥ 4:1 for all celiotomies, with higher ratio in 2 × 2 SBSI celiotomies (5.07 ± 0.49, range 4–5.77), compared to 5 × 5 SBSI celiotomies (4.32 ± 0.22, range 4.00–4.76) (*P* < 0.0001). Time to complete celiotomy closure was 8.57 ± 1.57 min (range 5.98–11. 28) for 2 × 2 SBSI celiotomies, and 5.02 ± 0.94 min (range 3.43–6.2) for 5 × 5 SBSI celiotomies. Surgical time was significantly longer for small SBSI celiotomies (*P* < 0.0001).

There was a moderate correlation between thickness of linea alba with BW (r = 0.34, with P = 0.005), and between thickness of linea alba with sex (*r* = 0.389, with *P* = 0.001). There was a moderate but non- significant correlation between BW with sex (*r* = 0.32, with *P* = 0.07). No correlation was observed between width of linea alba with BW (*r* = 0.19, with *P* = 0.3), width of linea alba with sex (*r* = 0.039, with *P* = 0.83), or width of linea alba and bursting strength (*r* = 0.024, with *P* = 0.895). There was a moderate correlation between thickness of linea alba with bursting strength (*r* = 0.51, with *P* = 0.001), BW with bursting strength (*r* = 0.40, with *P* = 0.02), and sex with bursting strength (*r* = 0.56, with *P* = 0.01).

### Bursting Strength

**Figure 3:**
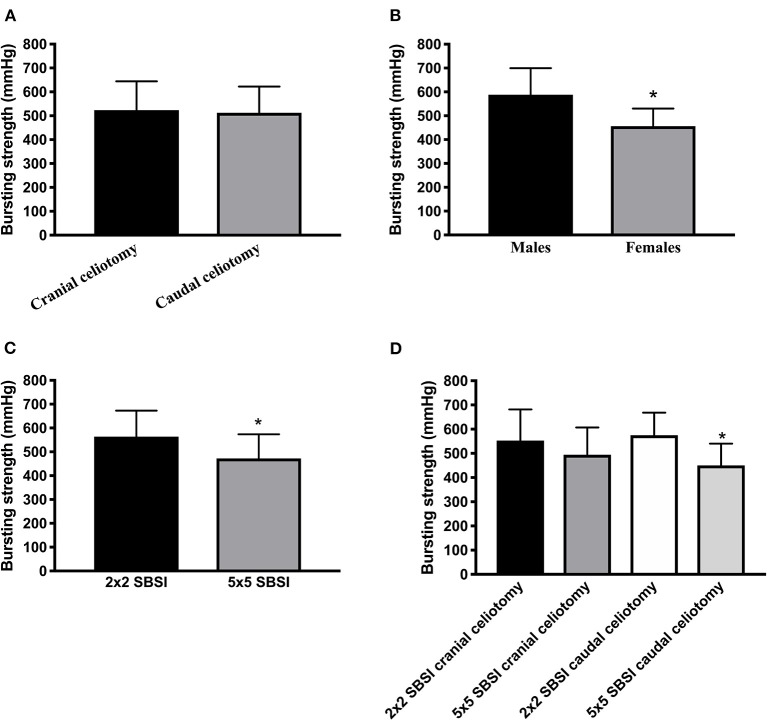
**(A)** Bursting strength of cranial and caudal celiotomies. **(B)** Bursting strength of males and females. **(C)** Bursting strength for small and large SBSI. **(D)** Bursting strength for celiotomy location and SBSI. *Statistical significant *P* < 0.05.

Bursting strength for the 32 specimens ranged from 327 to 770 mm Hg (518.22 ± 113.49). Mean ± SD bursting strength for cranial and caudal celiotomies was 523.81 ± 120.72 mm Hg (range 378–770), and 512.62 ± 109.44 mm Hg (range 327–704), respectively. No differences in bursting strength were observed between cranial and caudal celiotomies (*P* = 0.78) (Figure 3A).

Bursting strength (mean ± SD) for males was 578.9 ± 112.8 mm Hg (range 378–770), and 463 ± 86.79 mm Hg (range 327–704) for females. Bursting strength was higher for males compared to females (*P* = 0.003) (Figure 3B). Bursting strength for neuter status was not calculated due to low numbers of entire females and castrated males.

Bursting strength was 563.94 ± 109.31 mm Hg (range 411–770) for small 2 × 2 SBSI celiotomies, and 472.50 ± 101.04 mm Hg (range 327–706) for large 5 × 5 SBSI celiotomies. Bursting strength was higher for small SBSI compared to large SBSI celiotomies (*P* = 0.02) (Figure 3C).

Bursting strength was 553.1 ± 128.7 mm Hg (range 411–770) for small 2 × 2 SBSI cranial celiotomies, 574.8 ± 93.61 mm Hg (range 449–704) for small SBSI caudal celiotomies, 494.5 ± 112.7 mm Hg (range 378–706) for large 5 × 5 SBSI cranial celiotomies, and 450.5 ± 89.89 mm Hg (range 327–593) for large SBSI caudal celiotomies. Bursting strength for small SBSI caudal celiotomies was greater compared to large SBSI caudal celiotomies (*P* = 0.01). No other significant differences were observed between groups.

In the mixed model linear analysis, location of celiotomy did not have an effect on bursting strength (*P* = 0.34). Once this variable was removed from the statistical model (stepwise regression anaysis), SBSI showed significance with decreased bursting strength for large 5 × 5 SBSI celiotomies (*P* = 0.003). Once-way-ANOVA for SBSI showed decreased bursting strength for large SBSI caudal celiotomies compared to large SBSI cranial, and small SBSI cranial and caudal celiotomies (*P* = 0.02) (Figure 3D).

Sex, BW, and SBSI accounted for 61.5% of variability in bursting strength in the regression analysis (*P* = 0.005).

### Mode of Failure

**Figure 4:**
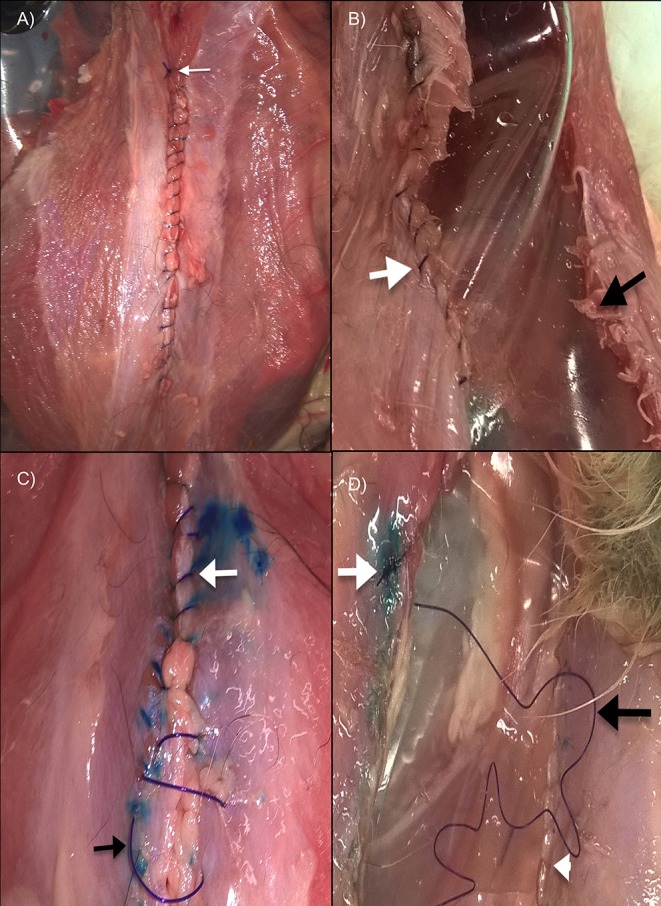
**(A)** Failure distant from celiotomy closure in a 2 × 2 SBSI cranial celiotomy. White arrow is at the suture line. **(B)** Failure by fascial tearing in a 2 × 2 SBSI cranial celiotomy. Notice cracks at the site of needle penetration (black arrow) with the suture line remaining intact (white arrow). **(C)** Failure by slacking (loss of suture line tension) at the caudal part of celiotomy closure (black arrow) while the cranial part of the closure (white arrow) still remains intact. **(D)** Same specimen at the end of the test showing as pressure increases complete loss of suture line tension occurs (black arrow) with the knots remaining intact (white arrow). White arrowhead is at the sites of needle penetration.

Twenty five specimens failed distant from celiotomy site (Figure 4A). Six of these 25 failed at the external oblique muscle immediately caudal to the costal arch (Figure 4A), 10 of 25 at the lumbar portion of the external oblique muscle, and 9 of 25 at the musculotendinous part of the external oblique muscle immediately cranial to the prepubic tendon. No differences were observed between right and left side failure for these 25 specimens.

Seven specimens failed at the celiotomy site. Four of these 7 failed by fascial tearing at the site of needle penetration (Figure 4B), while 3 of 7 failed by loss of suture line tension at the caudal part of the closure (Figure 4C) followed by separation of the wound edges as the pressure increased (Figure 4D), with the knots remaining intact. No breakage or failure of the knot occurred in any of the specimens.

Of the 16 small SBSI celiotomies, 14 failed distant from celiotomy site (7 cranial, 7 caudal celiotomies) and 2 by fascial tearing (1 cranial, 1 caudal). Of the 16 large SBSI celiotomies, 11 failed distant from celiotomy site (6 cranial, 5 caudal), 2 failed by fascial tearing (all cranial celiotomies), and 3 by loss of suture line tension (all caudal celiotomies).

Mean bursting strength for specimens that failed distant from celiotomy closure was 519 ± 123 mm Hg (range 302–770), while for specimens that failed at the celiotomy site was 510 ± 98 mm Hg (range 397–706). No differences in bursting strength were observed between these two types of failure (*P* = 0.85). Mean bursting strength for specimens that failed at the external oblique muscle caudal to the costal arch was 528.6 ± 125.7 mm Hg (range 327–694), 535 ± 107.2 mm Hg (range 411–706) for specimens that failed at the lumbar portion of the external oblique muscle, 534.4 ± 154.2 mm Hg (range 352–770) for specimens that failed at the musculotendinous part of the external oblique muscle cranial to the prepubic tendon, 451.8 ± 39.89 mm Hg (range 397–486) for failure by fascial tearing at the site of needle penetration, and 483.7 ± 38.07 mm Hg (range 447–523) for failure by loss of suture line tension. No differences in bursting strength were observed between the different modes of failure in the specimens (*P* = 0.74).

## Discussion

### Cranial vs. Caudal Celiotomies

We found no difference in bursting strength between cranial and caudal celiotomies, and our first hypothesis was rejected. Similar results have been reported in humans (20). This finding is in contrast to a previous biomechanical study in feline cadavers where the tensile strength of 3 areas of the ventral abdominal wall were compared (13). In that study, the postumbilical region was weaker than the other areas of the ventral abdominal wall (13). In our current study and under the loading conditions we used, the caudal abdomen, which contained the postumbilical region, better resisted failure. Therefore, it appears that the addition of the internal rectus sheath in the cranial abdomen (14, 15) does not provide any biomechanical advantage when cranial and caudal coeliotomy closure are compared. In the tensile strength study (13), the postumbilical region was selected because it would be the typical incision site for elective ovariohysterectomy in cats. However, in our current study regions are defined differently, making direct comparision of results impossible.

### Small vs. Large Suture-Bite-Stitch Interval Techniques

Small 2 × 2 SBSI celiotomies had greater bursting strength compared to large 5 × 5 SBSI celiotomies, particularly in caudal celiotomies. In the mixed model analysis adjusted for BW, sex, thickness and width of linea alba, large SBSI celiotomies had lower bursting strength compared to small SBSI celiotomies. These results support our hypothesis that small SBSI technique is biomechanically superior to large SBSI technique for celiotomy closure in adult cat cadavers. In people, advantages of the small bite technique include reduced incidence of surgical site infection, better distribution of tension across the celiotomy closure, and reduced incidence of incisional hernia (7–11).

The biomechanical advantages of the small bite technique were also reported in an experimental study in laboratory rats which concluded the small bite technique does not weaken the strength of wound closure or reduce the tissue holding capacity of the stitches as long as the SL:WL is ≥ 4:1 (19). A minimum SL:WL ≥ 4:1 is recommended in people to reduce the incidence of postsurgical incisional hernia (10, 21). In addition, the 4 to 1 rule is particularly important for the mechanical strength of the closure as a 30% lengthening in the surgical wound may be expected during the initial postoperative period (18). In our study, the SL: WL was ≥ 4:1 for all celiotomies, and the number of stitches using the small SBSI technique was nearly twice the number of stitches with the large SBSI. The higher bursting strength of the small SBSI celiotomies is likely because tension is distributed to more stitches within the suture line. The main disadvantage of the small SBSI was the longer time required to complete wound closure. In people, a 4 min increase in surgical time was reported using small SBSI (11). In our study, great care was taken to ensure the suture penetrated the points marked in the fascia, which increased the time to complete the celiotomy closure; however, we observed surgical time gradually decreased with practice.

### Males vs. Females

Bursting strength was greater in males than in females, as observed by the moderate correlation between sex and BW with thickness of the linea alba. Although the correlation between BW and sex was not significant, there was a trend for males to weigh more and have thicker linea alba. In people, factors such as age and sex may effect the composition of collagen fibers of the linea alba and rectus sheath, and potentially the biomechanical properties of the ventral abdominal wall (22–24). Sex differences in people include a higher elasticity of the ventral abdominal wall in women compared to men, with different load to failure in horizontal, vertical and oblique direction (24), and less compliance of the ventral abdomen in transverse direction in women compared to men, particularly in the infraumbilical region (23). In our study, males and females were grouped without considering neutering status. It is unknown if neutering may have an effect on the bursting strength of the linea alba in cats.

### Mode of Failure

No significant differences in bursting strength were observed between the modes of failure described; however, different modes of failure were recorded, which may have clinical importance. The most common mode of failure we observed was distant from the celiotomy site, which is consistent with studies in rats and horses (19, 25). Rupture of the abdominal wall away from the suture line would seem to indicate that under our loading conditions, the strength of the suture line exceeds that of the intact abdominal wall.

Failure by loss of suture line tension followed by separation of the wound edges, in the absence of breakage of suture material or failure of the knots, was only observed in caudal celiotomies closed with a large SBSI. This mode of failure was also described in a previous study evaluating the tensile strength of discrete segments of the feline ventral abdominal wall when a large SBSI was used to close celiotomies (13). In people a large SBSI has been associated with cutting or compression of soft tissue included in the bite, leading to separation of the edges of the incision without failure of the suture material (9–11). This mode of failure has been described as “slacking” and is only observed with the use of a large SBSI technique (10). The mode of failure observed in some of our large SBSI caudal celiotomies resembles this form of failure. Although not all of the large SBSI caudal celiotomies failed by slacking, this mode of failure was not observed in any of the small SBSI celiotomies. In addition, slacking was only seen with large SBSI in our previous study where we evaluated the tensile strength of ventral abdominal wall closure (13). Using a small SBSI overcomes the “slacking” effect, as less soft tissue is included in each bite and the load is spread over more sutures (10), and explains why we did not observe this mode of falure in the small SBSI celiotomies.

Failure by fascial tearing at the site of needle penetration has been previously reported as a late complication of celiotomy closure in people [26, 27, p. 243–244). This type of failure is known as “buttonhole” incisional hernia, and has been mainly associated with the use of non-absorbable suture material (26). It is believed that the slight but repeated movement of the non- absorbable suture material in relation to the abdominal wall throughout time may have a sawing effect of a cheese wire (26). However, buttonhole hernias have also been described when absorbable suture materials are used (27). According to this author, this type of failure may occur if an excessive tension is applied in the suture material during closure which can result in a mechanical failure similar to an early postoperative wound dehiscence (27). The use of an optimal SL:WL and avoidance of tension during closure have been recommended to prevent this type of complication in people (26, 27). Although we did not measure tension at the suture line in our celiotomies, our closure techniques caused no tension at the tissues and only aimed to appose the wound edges. We also used an optimal SL:WL, a long lasting absorbable monofilament suture material and a round body atraumatic small needle. Thus, the excessive tension caused at the suture line by our testing apparatus were likely responsible for this mode of failure, which resembles the buttonhole incisional hernias described in people.

### Characteristics of Linea Alba

The linea alba was widest at the umbilicus and narrowed both cranially and caudally in all specimens; however, no correlation was observed between linea alba width and bursting strength. Width of linea alba did not influence suture placement in our celiotomies, as suture bites were measured from the lateral edges of the linea alba. Based on human cadaveric (16) and laboratory animal studies (19, 28), the strength of a celiotomy closure markedly increases when sutures engage the area between the linea alba and the rectus sheath or transition zone, and this technique was consistently used throughout the study.

Thickness of linea alba decreased gradually from cranial to caudal in all the specimens; however it did not have an effect on bursting strength when cranial and caudal celiotomies were compared. This is in contrast to an experimental study in rats, where the cranial third of the abdominal wall had lower bursting strength (28), and an experimental study in cats, where the thinner postumbilical region had lower load to failure (13).

### Full-Thickness vs. Fascia-Only Closure

Inclusion of muscle rather than fascia during celiotomy closure is considered a technical error that may cause incisional hernia in small animals (1). Our celiotomies were closed with a fascia-only closure technique, with sutures engaging only the external sheath of the rectus abdominis muscle, which has been shown to be the primary strength-holding layer for celiotomy closure in dogs (29). Fascia-only closure is biomechanically equivalent to full-thickness closure in cats (13) and could potentially reduce post-operative pain, which has been shown to increase tension in the celiotomy closure after surgery in humans (10).

### Selection of Suture Material, Suture Size, and Needle Type

Our celiotomies were all closed with polydioxanone, a monofilament, synthetic, absorbable suture material with high biomehanical strength (30) and long *in vivo* strength retention [(31), p. 180–191]. Polydioxanone has been recommended for celiotomy closure, particularly in patients where incisional healing could be delayed or a major risk for postsurgical infection of the incision, exists (8, 10). This suture material has also been tested in several studies (7, 9, 10, 13, 19, 25, 30), and has been recommended for celiotomy closure in domestic cats ((32), p. 105–116).

Selection of a particular diameter of suture material depends on the patient size, the tissue being sutured, and the potential healing of the tissue (33, 34). Two metric (3- 0 USP) suture diameter is commonly recommended for celiotomy closure in domestic cats (32). The use of larger diameter suture materials may provide higher breaking strength, and greater pull-out force when compared to suture material with a smaller diameter (30, 35). However, excessively large diameter sutures may lead to more foreign material at the surgical site and decreased knot security (33, 34). It is likely that the use of larger diameter suture would have influenced our results, however, the diameter of the suture material used in this study was selected as it is commonly used in domestic cats.

We selected a 26 mm tapered semicircular needle for celiotomy closure as this needle is recommended for muscle and fascial closure (31), and is commonly used in clinical cases (32). It is possible that a smaller size needle would have reduced the number of specimens that failed by tearing of the fascia. The use of smaller needles reduces damage to the tissues at the site of needle penetration resulting in tissue that is more resistant to tearing (7–11).

### Other SBSI Combinations

In small animals, including domestic cats, the recommended distance from the wound edges where sutures are placed (suture bite) and the distance between stitches (suture interval) for celiotomy closure are based on clinical experience (5, 12). We evaluated 2 SBSI techniques to determine if there was any biomechanical benefit to using a “small” vs. a “large” SBSI technique, as proposed in people (7, 8, 10, 11). The tensile strength of these SBSI techniques in the linea alba of cats was previously evaluated (13). In this study we evaluated the bursting pressure which we believe provides a better understanding of the mechanical properties of these celiotomy closure techniques in cats. Biomechanical testing has also been performed on a variety of suture patterns where suture bite and stitch interval were varied independently (36). This study showed that the use of smaller bites (suture bites) and larger bite widths (stitch interval) provided a stronger celiotomy closure when compared to other combinations (36). In a further study, the combination of a small suture bite and a large stitch interval significantly increased the suture pull- out force (35). Although these 2 studies suggest that other techniques offer a biomechanical advantage, the testing apparatus and the species used differ from this study, preventing direct comparison of the results.

## Conclusions and Limitations

The mean bursting strength in our specimens was higher compared to laboratory animals (19, 28) and horses (25). The higher pressures obtained in our specimens, particularly compared to horses, may be related to the collagen composition of the linea alba and rectus sheath in domestic cats, as postulated in humans (22, 24). The variation in the collagen composition and distribution in this area of the ventral abdominal wall in this species may also explain the inter-, and intra-group variation in bursting strength observed.

The methodology we used has been previously described in experimental studies in rats (19, 28), horses (25), and humans (20, 37); however, inflation rates differ between studies and results cannot be directly compared. The testing apparatus we used mimics the effect of increased intraabdominal pressure (IAP) and closely reproduces three-dimensional forces acting on the incision *in vivo* (37). The single cycle to failure used in this and other studies (19, 25, 28) is unlikely to occur *in vivo*, and is a limitation of this study. Different results would likely have been obtained using cyclic fatigue, which may more closely mimic a clinical scenario.

The use of material harvested from cadavers that were frozen and thawed at room temperature, and thereafter preserved in a saline bath under refrigeration is another limitation of this study. However, this protocol should not affect the biomechanical properties of the tissue making the specimens useful for analysis (20).

The natural variation that exists when biological tissue is tested, including interindividual and intraindividual variation, as reported in humans (22, 24), is also a limitation of this study. In a cadaveric study suture degradation, surgical site infection, prolonged inflammation, suture failure during cyclic and repetitive strain, and other patient-dependent factors that may cause incisional herniation cannot be evaluated (1, 9–11).

The ideal SBSI in dogs and cats is unknown. It has been stated that celiotomy closure should approximate the linea alba without allowing abdominal viscera to protrude between stitches and should not weaken the fascia or compromise the blood supply with multiple perforations (12). The small 2 × 2 SBSI was superior to the large 5 × 5 SBSI; however, the pressures we applied to cause failure of the celiotomies were supraphysiological compared to the normal IAP reported in healthy cats (38). For this reason, both SBSI techniques may be applicable in clinical cases.

Studies such as this one improve our understanding of the mechanical behavior of celiotomy closure and provide scientific evidence to reduce technical errors that can lead to incisional hernia. We caution that the findings of our study cannot be applied directly to clinical cases, and prospective clinical trials are required to determine optimal closure technique for ventral midline celiotomy in cats.

## Data Availability Statement

All datasets generated for this study are included in the article/supplementary material.

## Ethics Statement

The animal study was reviewed and approved by UCD Animal Ethics Committee (ref. AREC P - 12-77- Reina).

## Author Contributions

FR performed the experimental study under the supervision of BK. FR, JM, and BK contributed to the development of the final version of this manuscript.

## Conflict of Interest

The authors declare that the research was conducted in the absence of any commercial or financial relationships that could be construed as a potential conflict of interest.

## Abbreviations

BW: Body weight (kg)
CdA: caudal abdomen
CrA: cranial abdomen
IAP: intraabdominal pressure
SBSI: suture bite to stitch interval
SL:WL: suture length to wound length ratio
U: umbilicus.
